# An Improved Deepfake Detection Approach Using Hybrid Architecture Based on EfficientNet and Vision Transformer

**DOI:** 10.3390/jimaging12090427

**Published:** 2026-09-09

**Authors:** Omar Banimelhem, Abeer O. Alsharu

**Affiliations:** Department of Network Engineering and Security, Jordan University of Science and Technology, Irbid 22110, Jordan; aoalsharu@just.edu.jo

**Keywords:** deepfake detection, Efficient-ViT, hybrid model, transformer networks, deep learning

## Abstract

The rapid spread of deepfake content poses a significant threat to the credibility of digital multimedia, creating an urgent need for accurate and robust detection methods. This paper proposes a hybrid deep learning framework that combines EfficientNet-B0 and Vision Transformer (ViT-B/16) through feature concatenation to exploit both local spatial representations and global contextual dependencies for image-level deepfake detection. The proposed model was trained and evaluated on the deepfake and real images dataset. Experimental results demonstrate that the proposed framework achieves an accuracy of 0.9870, an F1-score of 0.9871, and an AUC of 0.9990. Additional cross-dataset evaluation on the CelebDF-v2 image dataset and robustness experiments under common image degradations further demonstrates the strong generalization capability and practical applicability of the proposed approach. These results confirm that integrating CNN-based and Transformer-based feature extraction provides an effective and reliable solution for deepfake image detection.

## 1. Introduction

The rapid spread of deepfake technology has made it possible for individuals with minimal technical skills to alter visual content using commonly available digital applications [1]. As this technology becomes more accessible, concerns have intensified about its potential misuse in harmful or unethical ways [2]. The word “Deepfake,” which combines the terms “Deep Learning (DL)” and “Fake,” describes photorealistic pictures or videos produced by a Deep Neural Network (DNN) that appear real to the human eye [3].

In addition to deepfake pornography, deepfake technology can be used maliciously for several illegal and unethical activities, including identity theft, financial fraud, political manipulation, cyberbullying, disinformation campaigns, impersonation scams, cyber extortion, and the spread of misleading content that may influence public opinion or incite social instability [4].

**Figure 1:**
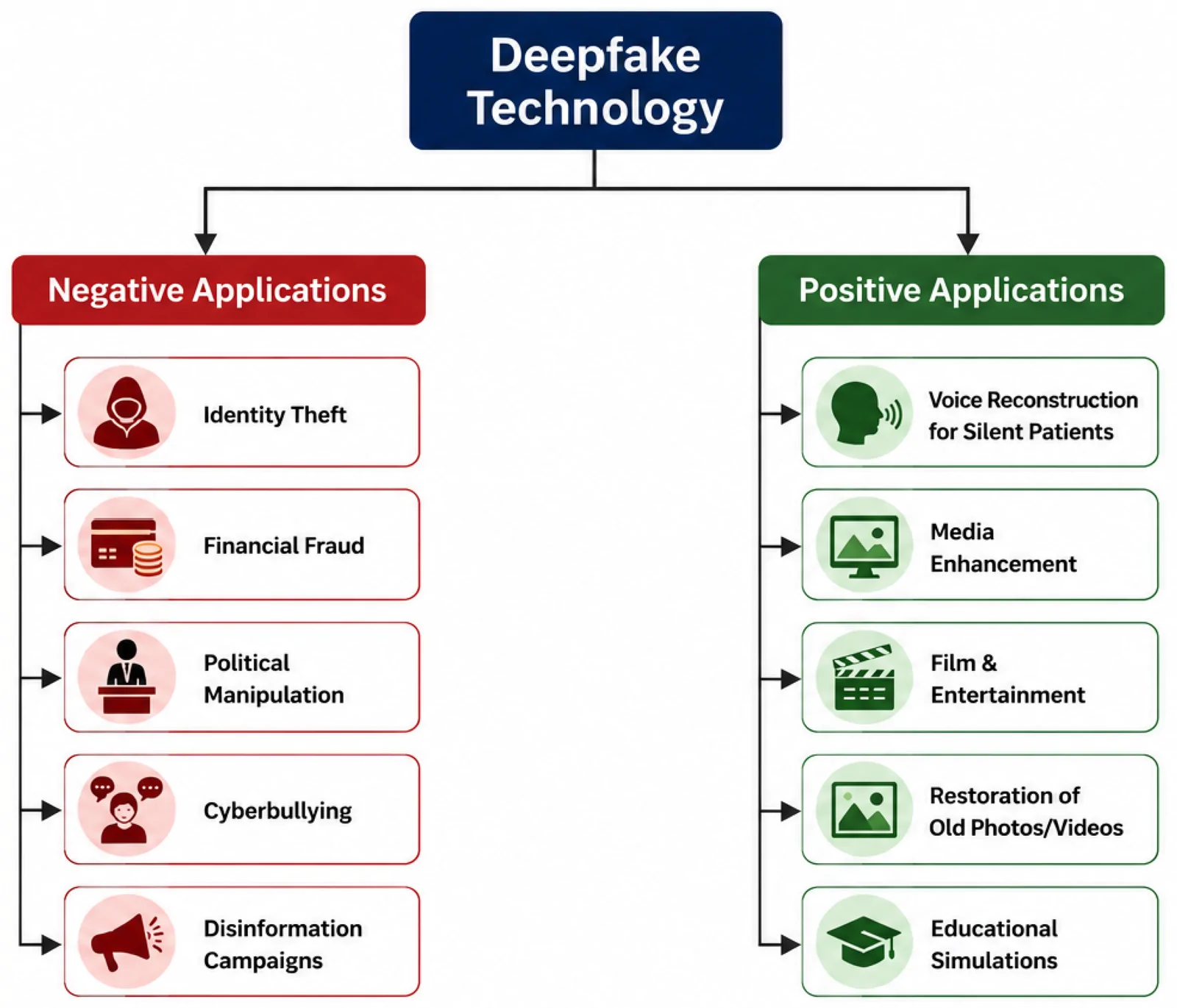
Common positive and negative applications of deepfake technology.

Deepfake-related crimes continue to increase rapidly on different digital platforms. However, despite these negative impacts, deepfake technology can also provide beneficial applications in several domains. Positive uses include voice reconstruction for silent patients, media enhancement, restoration of old photos and videos, educational simulations, and applications in the film and entertainment industry. Although these applications may support creativity, accessibility, and digital media development, the misuse of deepfake technology still represents a significant threat to individuals and institutions. Figure 1 illustrates both the positive and negative applications of deepfake technology across different domains.

Deepfake content is mostly produced using deep learning techniques, especially generative adversarial networks (GANs) and autoencoder models [5]. To produce synthetic images and movies, the algorithms of these techniques are trained on enormous datasets [6]. As more data becomes available, these algorithms’ capacity to produce compelling content increases dramatically [7].

Based on the above, celebrities, politicians, and prominent figures are the biggest victims of deepfake technology due to the abundance of images available to them [8]. This technology, through its convincing methods, can mislead the public, influence public opinion, incite political and religious conflicts, and destabilize the economy of societies. For these reasons, many deep learning-based detection techniques have been developed to combat this threat [9]. Convolutional neural networks (CNNs) are frequently utilized because of their ability to detect tiny distortions caused by image processing, as well as their ability to infer local and spatial properties [10]. Despite their effectiveness, they ignore long-term dependencies and contextual information included in the images.

Transformer-based models, such the Vision Transformer (ViT), have lately shown promising results in detecting deepfakes by using self-attention techniques to simulate global linkages. Despite their advantages, they need large datasets and only perform mediocrely when employed alone. Combining CNNs with transformers has become a popular strategy for enhancing performance due to their complementing qualities [11].

This paper offers a hybrid model that combines EfficientNet, a CNN-based architecture known for its efficiency and strong feature extraction capability, with a Vision Transformer to utilize both local and global representations. The retrieved features from both models are combined to create a more reliable and accurate deepfake detection system.

In conclusion, the main contributions of this work are summarized as follows:

1. A hybrid deep learning framework is proposed for image-based deepfake detection by integrating EfficientNet-B0 and Vision Transformer (ViT-B/16) as parallel backbone networks within a unified architecture.
2. The proposed architecture combines local feature extraction capabilities provided by EfficientNet with the global contextual representation learning of ViT, enabling more effective detection of manipulated facial images.
3. A feature-level concatenation strategy is employed to fuse the complementary feature vectors extracted independently by EfficientNet-B0 and ViT-B/16 before the final classification stage.
4. Extensive experiments are conducted using multiple evaluation metrics, including Accuracy, Precision, Recall, F1-score, and AUC, to validate the effectiveness of the proposed approach.
5. The proposed hybrid model outperforms the individual EfficientNet and ViT architectures and achieves competitive performance compared with recently reported state-of-the-art deepfake detection methods.

The novelty of the proposed framework lies in employing EfficientNet-B0 and ViT-B/16 as two parallel backbone networks and integrating their complementary feature representations through feature-level concatenation within a unified hybrid architecture for image-based deepfake detection. Unlike some existing hybrid CNN–Transformer approaches, where convolutional features are embedded into transformer architectures or additional architectural modifications are introduced, the proposed framework performs parallel feature extraction using both backbone networks and fuses the extracted representations only before the final classification stage. This design enables the model to jointly exploit fine-grained local artifacts and global contextual information while maintaining a simple and effective architecture for deepfake image classification.

This paper is organized as follows: Section 2 presents the related work on deepfake generation and detection. Section 3 describes the proposed methodology. Section 4 presents the experimental results obtained from the proposed approach. Section 5 discusses and analyzes the obtained results. Finally, Section 6 concludes the paper and outlines directions for future work.

## 2. Related Work

Existing deepfake detection studies can broadly be divided into image/frame-level approaches and video-level approaches. Image/frame-level methods primarily analyze spatial artifacts within individual facial images or frames, whereas video-level approaches additionally exploit temporal information, recurrent modeling, optical flow, or frame-level aggregation to make a video-level decision. This distinction is important when comparing reported results, since video-level methods benefit from temporal information that is not available to image-level detectors.

To enhance detection capabilities and provide more comprehensible findings, deepfake detection now relies on sophisticated deep learning techniques. In this field of study, conventional convolutional neural networks (CNNs) have been crucial. This section provides an overview of relevant research on deepfake detection.

Researchers in [12] developed a methodology for a deepfake detection approach that examines head position discrepancies and highlights that edited videos frequently lose authentic three-dimensional head movements. To detect deepfakes, the authors in [13] devised an approach that focuses on identifying face warping artifacts, which reveal abnormal distortions caused by face swapping procedures. The authors in [14] proposed a CNN-based algorithm for detecting convolutional traces in fake images and proposed a forensic method for detecting deepfakes.

Recent research has looked at hybrid systems created by combining CNNs with Vision Transformers (ViTs) to enhance detection performance. The model shown in [15] combines EfficientNet with ViTs to increase accuracy by using global attention processes from transformers and local feature extraction from CNNs. Accuracy has reached 0.95. The M2TR method, proposed in [16], improves deepfake identification on large datasets by fusing spatial and temporal properties using multi-modal multi-scale transformers. Accuracy has reached 0.95. The interpretability of deepfake detection algorithms has been at the forefront of the field in recent years.

Shapley Additive Explanations (SHAP) was used in [17] to examine the decision-making process of deepfake detectors, emphasizing the need for explainable AI in fostering confidence and trust in forensic settings. The wider social implications of deepfakes were also covered in [18], highlighting the importance of effective detection systems in stopping the growing manipulation of artificial intelligence-generated synthetic content.

When it comes to identifying deepfakes, recurrent neural networks (RNNs) have shown promise, particularly when it comes to analyzing temporal discrepancies. While the researchers in [19] looked at recurrent convolutional techniques for identifying changes in face regions, the researchers in [20] suggested a two-branch recurrent network to assess phony areas within video information. The authors proved that sequential models improve deepfake detection by identifying timing-based discrepancies that fixed CNN techniques fail to detect.

The DFDC dataset was introduced in [21] as a sizable public benchmark that aids in the advancement of detection algorithm research. Rossler et al. [22] developed the FaceForensics++ dataset using standardized deepfake identification tests for classifiers. Researchers think that detecting flaws may be resolved by hybrid deep learning frameworks that incorporate several frameworks. The authors in [23] proposed a sophisticated detection method that analyzes phony video movement patterns using CNN-LSTM algorithms and optical flow characteristics. The authors in [24] investigated how hierarchical characteristics that differentiated between authentic and fraudulent information content might be used by capsule networks to identify phony photographs. In order to improve the recognition of tiny alterations made by AI generation, the team of [25] created a deepfake architecture that blends MLP and LSTM. Their research shows that combining geographical, temporal, and sequential analysis maximizes the accuracy of deepfake detection.

The increasing need for technologically sophisticated and scalable solutions is proof that deepfake detection technology is still developing. The combination of CNNs, transformers, RNNs, and hybrid techniques has significantly increased the accuracy of AI detection, opening up new avenues for future study on opposing artificial intelligence-altered synthetic media.

Despite the progress of these approaches, an important methodological gap remains. Image-level methods based on CNNs are effective at capturing local manipulation artifacts, while Transformer-based models can capture broader contextual relationships. In contrast, several hybrid and recurrent approaches in the literature incorporate temporal information and are primarily designed for video-level detection. Therefore, their results are not directly comparable with an image-level framework that operates on individual facial images. The present study addresses this gap by investigating an image-level hybrid architecture that combines EfficientNet-B0 and ViT-B/16 through feature fusion, aiming to exploit complementary local and global visual representations without relying on temporal information. Table 1 presents a summary of the related work discussed in this section.

## 3. Methodology

This section describes the methods and approaches utilized to develop the proposed hybrid deepfake detection framework. The proposed model combines convolutional neural networks and transformer-based architectures to improve the detection of manipulated facial images. It should be noted that the proposed framework is designed specifically for image-level deepfake detection. Unlike video-based approaches that exploit temporal information, optical flow, or motion consistency across consecutive frames, the proposed method processes and classifies each image independently based solely on its spatial visual features.

The dataset and image preprocessing procedures are described in Section 3.1. Section 3.2 presents the proposed hybrid framework. The feature extraction process based on the EfficientNet-B0 and Vision Transformer (ViT-B/16) models is described in Section 3.3. Finally, Section 3.4 describes the feature fusion and classification process.

### 3.1. Dataset and Preprocessing

The experiments were conducted using the publicly available deepfake and Real Images dataset available on the Kaggle platform [26], which was derived from the Zenodo repository. The dataset contains 190,335 facial images, including both authentic (real) and manipulated (fake) images generated using various deepfake manipulation techniques. All images are provided in JPEG format with a resolution of 256×256 pixels.

The dataset is organized into predefined training, validation, and testing subsets provided by the dataset authors. In this work, the original dataset partition was adopted without any modification. The dataset documentation does not explicitly specify whether the partition was performed at the image, identity, or source level. Therefore, the official dataset split was retained throughout all experiments. Table 2 shows the number of images and class distribution for each subset. The training set contains 140,002 images (70,001 real and 70,001 fake), the validation set contains 39,428 images (19,787 real and 19,641 fake), and the testing set contains 10,905 images (5413 real and 5492 fake).

**Figure 2:**
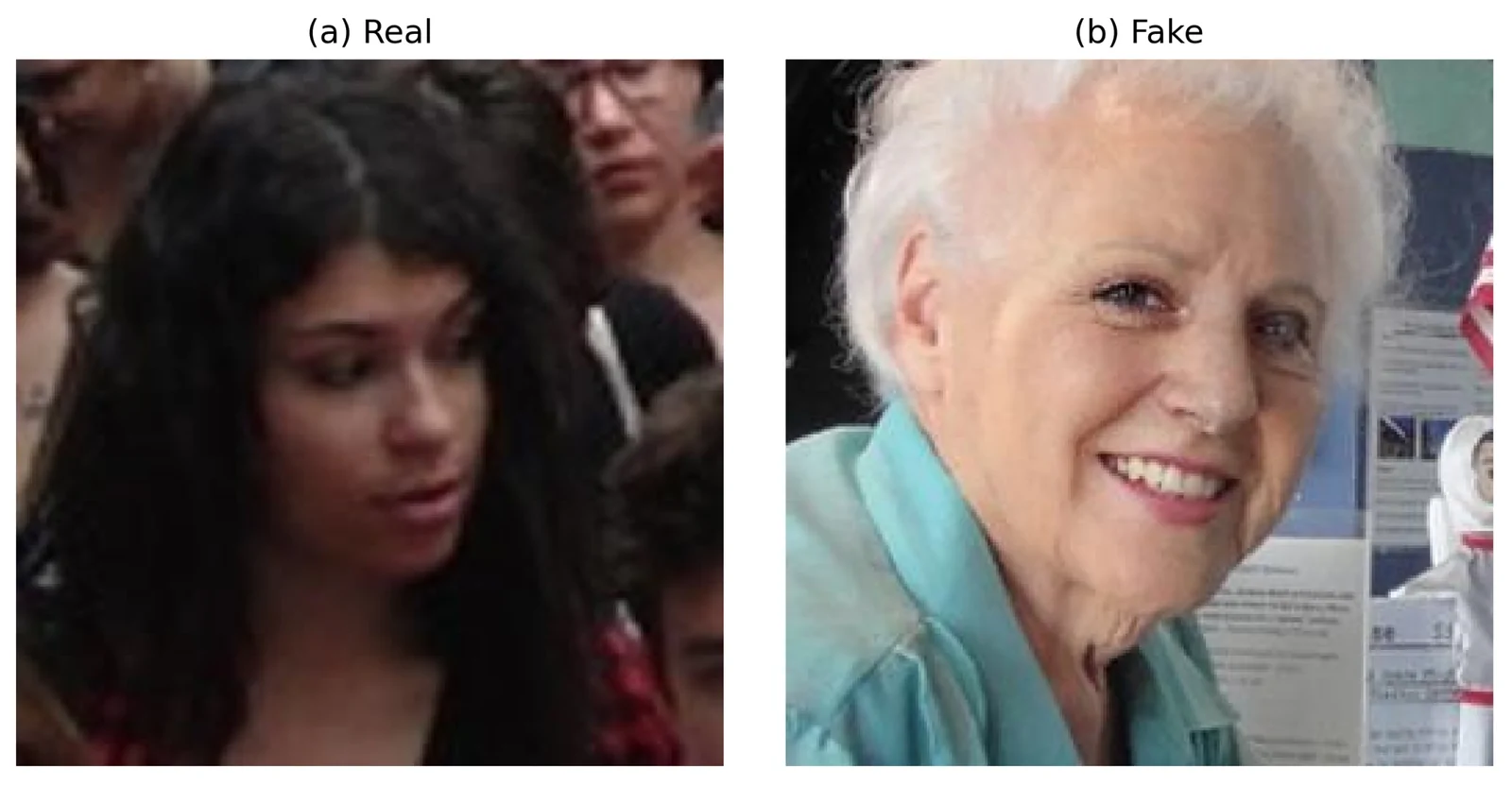
Representative examples of authentic (real) and manipulated (fake) facial images from the dataset.

**Figure 3:**
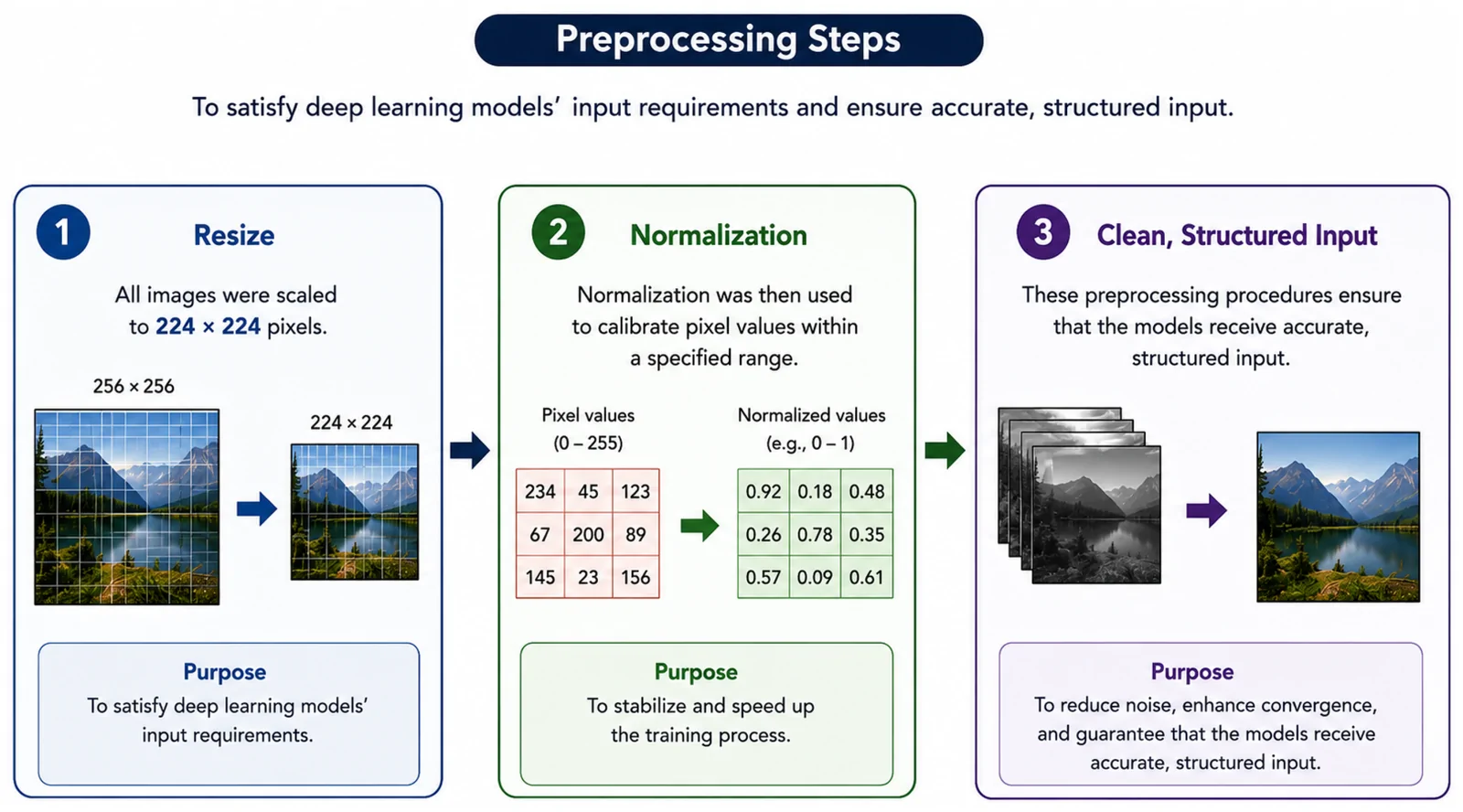
Preprocessing steps applied to the dataset.

To satisfy the input requirements of the proposed EfficientNet–ViT hybrid framework, all images were resized from 256×256 to 224×224 pixels prior to training. Pixel values were then normalized to stabilize the optimization process and accelerate model convergence. These preprocessing steps reduce data variability and ensure consistent input to both backbone networks. Figure 2 shows representative examples of authentic (real) and manipulated (fake) facial images from the dataset, and Figure 3 illustrates the preprocessing pipeline applied to the dataset.

To verify the reliability of the evaluation protocol, a duplicate-image analysis was performed between the training and testing subsets. No duplicate images were identified between these two subsets, indicating that the reported test performance was obtained without direct data leakage.

### 3.2. Proposed Approach

**Figure 4:**
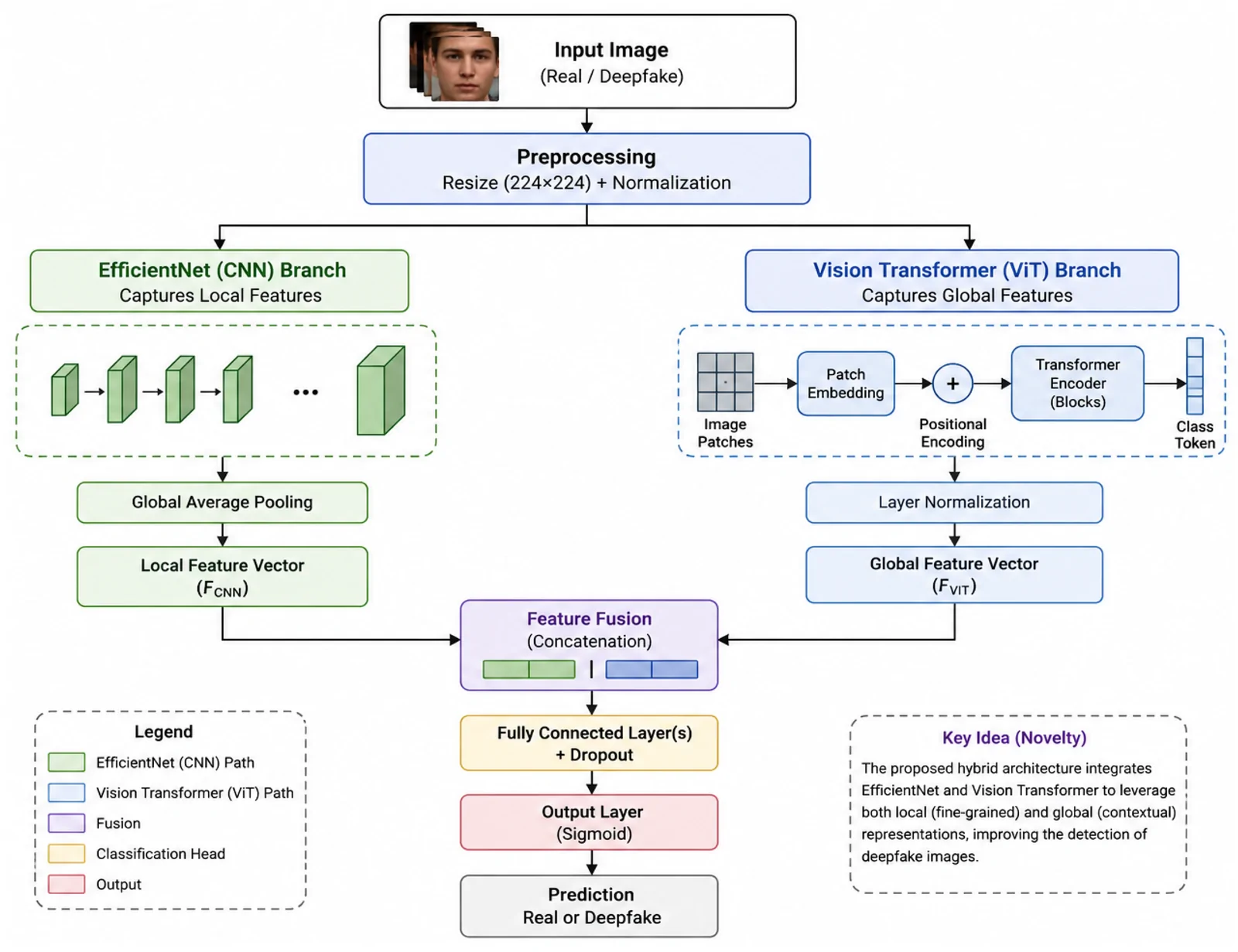
Flowchart of the proposed approach.

The proposed work is novel in that it combines EfficientNet and Vision Transformer (ViT) via a feature fusion mechanism, as opposed to conventional deepfake detection approaches that rely solely on convolutional neural networks or transformer-based architectures. EfficientNet captures fine-grained local artifacts caused during image modification, whereas ViT models global contextual relationships within facial photos. The incorporation of these complementary representations allows the proposed model to increase deepfake detection performance and robustness. Figure 4 illustrates the architecture of the proposed hybrid deepfake detection framework combining EfficientNet and Vision Transformer through feature fusion. Also, Algorithm 1 illustrates the steps of this approach:
**Algorithm 1** Proposed Hybrid Deepfake Detection Framework**Require:** Facial image *I***Ensure:** Predicted label $\hat{y}\in \left\{\mathrm{Real},\mathrm{Deepfake}\right\}$  1:  $I_{\mathrm{processed}}\leftarrow \mathrm{Resize}\left(I,224\times 224\right)$  2:  $I_{\mathrm{norm}}\leftarrow \mathrm{Normalize}\left(I_{\mathrm{processed}}\right)$  3:  {**Parallel Feature Extraction**}  4:  $F_{E}\leftarrow \mathrm{EfficientNet}-B0\left(I_{\mathrm{norm}}\right)$  5:  $F_{V}\leftarrow \mathrm{ViT}-B/16\left(I_{\mathrm{norm}}\right)$  6:  {**Feature Fusion**}  7:  $F_{H}\leftarrow \mathrm{Concatenate}\left(F_{E},F_{V}\right)$  8:  {**Classification**}  9:  $Z\leftarrow \mathrm{FullyConnected}(F_{H})$10:  Score←Softmax(Z)11:  **if** 
Score[Deepfake]>Score[Real]
 **then**12:    $\hat{y}\leftarrow \mathrm{Deepfake}$13:  **else**14:    $\hat{y}\leftarrow \mathrm{Real}$15:  **end if**16:  **return** 
$\hat{y}$

To improve the reproducibility of the proposed framework, the implementation details are shown in Table 3. The proposed framework was implemented using PyTorch v2.9.0+cu126 deep learning framework. EfficientNet-B0 and Vision Transformer (ViT-B/16), both initialized with ImageNet pretrained weights, were employed as the backbone networks. All input images were resized to 224×224 pixels and normalized prior to training. Both backbone networks were fully fine-tuned using the Adam optimizer with a learning rate of $1\times 10^{-4}$. The extracted feature vectors from EfficientNet-B0 and ViT-B/16 (1000 dimensions each) were concatenated into a 2000-dimensional fused representation, which was subsequently fed into a fully connected layer for binary classification. No additional data augmentation techniques were applied. Furthermore, the dataset consists of pre-cropped facial images; therefore, no separate face detection or face alignment stage was required.

### 3.3. Feature Extraction

**Figure 5:**
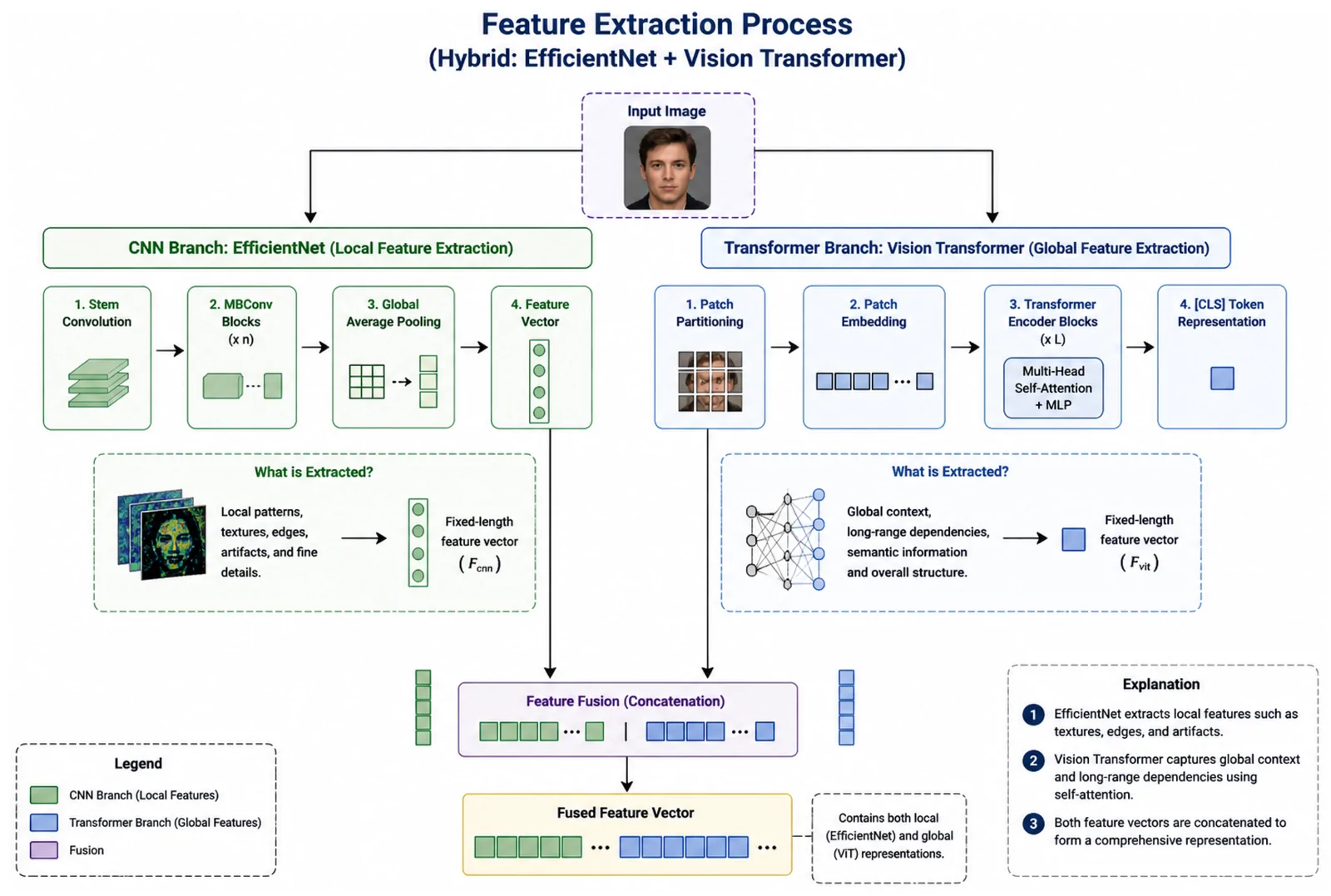
Feature extraction with EfficientNet and Vision Transformer.

We used two methods for feature extraction. First, we used the EfficientNet technology to capture local visual patterns and tiny aberrations that occurred during image processing. This method’s identified features assist in showing texture imperfections and small details. The second technique, which made use of self-attention mechanisms and the Vision Transformer (ViT) [27], was then used to depict general relationships within the visuals. This strategy aids in capturing long-term dependencies and contextual information that EfficientNet may be unable to discover on its own. Figure 5 illustrates the feature extraction stage using EfficientNet and Vision Transformer in parallel to capture both local and global features.

### 3.4. Feature Fusion and Classification

The outputs of EfficientNet and Vision Transformer are merged to create a single representation that includes both local and global information. After passing through completely connected layers, the outcome is categorized as either real or fake categories.

To optimize transfer learning and speed up convergence, all models are initialized and adjusted using pre-trained weights. A cross-entropy loss function was used to train each model, and the Adam optimizer was used for optimization. The techniques’ performance was assessed using a number of criteria, including accuracy, the F1 metric, and the area under the curve (AUC).

**Figure 6:**
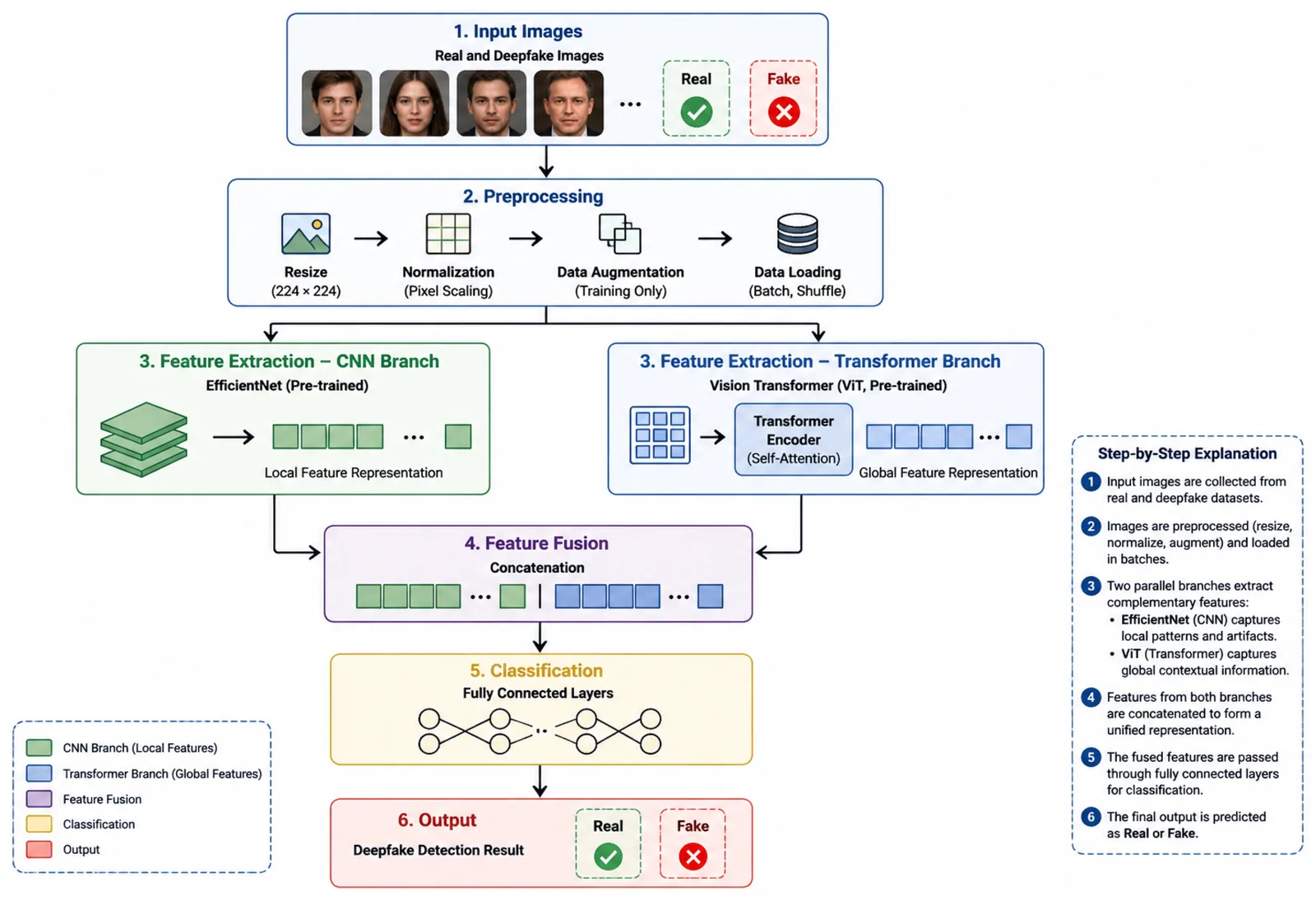
The overall architecture of the proposed hybrid model.

Figure 6 displays the proposed framework’s multi-stage pipeline, which begins with input images being preprocessed and then moves on to feature extraction via concurrent branches of EfficientNet and Vision Transformer. The retrieved features are then merged and applied to the final classification.

## 4. The Experimental Results

This section discusses the experimental configuration and the specific parameters employed to train and assess the suggested classification models. We also consider the evaluation metrics used to analyze the success of these models. Finally, we present and analyze the model’s results.

### 4.1. Environment Configuration

The proposed model was tested on the Kaggle platform, established by Google LLC (Mountain View, CA, USA). Kaggle is a cloud computing service that facilitates various professional projects in multiple programming domains, allowing us to execute the entire project using the Python language. This platform offers a extensive range of features and robust hardware resources. Table 4 provides a detailed summary of the work environment, which includes the software and hardware used in our research.

### 4.2. Model Hyper-Parameters

We conducted several experiments to determine the most effective hyperparameter configuration for the proposed models. Table 5 shows the selected hyperparameters with the best performance.

The proposed hybrid model was trained for 8 epochs using Adam optimizer with a learning rate of $1\times 10^{-4}$ and cross-entropy loss. No learning-rate scheduling was applied. The checkpoint achieving the highest validation accuracy during training was retained for the final evaluation.

### 4.3. Evaluation Measures

To evaluate the performance of the proposed deepfake detection model, standard evaluation metrics were utilized, including Accuracy, Precision, Recall, F1-score, and Area Under the ROC Curve (AUC). In the context of binary classification for deepfake detection, the underlying confusion matrix terms are defined as follows:

- True Positives (TP): The number of manipulated (fake) images correctly identified as fake by the model.
- True Negatives (TN): The number of authentic (real) images correctly identified as real by the model.
- False Positives (FP): The number of authentic (real) images incorrectly classified as manipulated (fake).
- False Negatives (FN): The number of manipulated (fake) images incorrectly classified as authentic (real).

Based on these foundational metrics, the overall quantitative evaluation measures are calculated as follows:

- Accuracy measure is one of the most famous metrics used in evaluating deep and machine learning models, is a measure of a model’s correctness, defined as the ratio of correct predictions to total predictions. The equation (see Equation (1)) is used to measure it:
  (1) $$Accuracy=\frac{\mathrm{TP}+\mathrm{TN}}{\mathrm{TP}+\mathrm{TN}+\mathrm{FP}+\mathrm{FN}}$$
- Precision: is defined as the ratio of correct positive predictions to total positive predictions, and it is used to calculate the positive predicted value. The equation (see Equation (2)) is used to measure it:
  (2) $$Precision=\frac{\mathrm{TP}}{\mathrm{TP}+\mathrm{FP}}$$
- Recall: Measures the model’s sensitivity by calculating the ratio of correctly identified positive cases to the total actual positive instances in the dataset. The equation (see Equation (3)) is used to measure it:
  (3) $$Recall=\frac{\mathrm{TP}}{\mathrm{TP}+\mathrm{FN}}$$
- F1-score: is defined as the harmonic mean of Precision and Recall. It provides a balanced evaluation of the trade-off between precision and sensitivity, particularly in scenarios involving class distribution imbalances. The equation (see Equation (4)) is used to measure it:
  (4) $$F1-\mathrm{score}=2\times \frac{\mathrm{Precision}\times \mathrm{Recall}}{\mathrm{Precision}+\mathrm{Recall}}$$
- The Area Under the Curve (AUC): assesses the model’s ability to differentiate between real and fake images. It measures the area under the receiver operating characteristic (ROC) curve.

## 5. Results and Discussions

The proposed models were evaluated using Accuracy, Precision, Recall, F1-score, and Area Under the ROC Curve (AUC) to provide a comprehensive assessment of their deepfake detection performance.

The EfficientNet model achieved an accuracy of 0.9754, demonstrating its effectiveness in detecting localized visual artifacts in manipulated images. The Vision Transformer (ViT) achieved an accuracy of 0.9619, which was slightly lower than that of EfficientNet, although it still showed strong classification performance with an AUC of 0.9936. The proposed Hybrid model outperformed both individual models, achieving an accuracy of 0.9870, an F1-score of 0.9871, and an AUC of 0.9990. These results highlight the advantage of combining CNN-based and Transformer-based feature extraction mechanisms within a unified framework, leading to improved deepfake detection performance.

The strength of the hybrid model is its ability to include the complementary data from both models. The ViT model concentrates on global dependencies between images, whereas the EfficientNet model only considers fine-grained details. When these two characteristics are combined, the hybrid model’s predictions become more precise and trustworthy. Additionally, the model’s capacity to discriminate between authentic and fraudulent images across a range of resolution criteria is demonstrated by the high AUC values. Overall, the experimental findings demonstrate that combining convolutional and transformer-based models leads to improved robustness and generalization in deepfake detection tasks.

**Figure 7:**
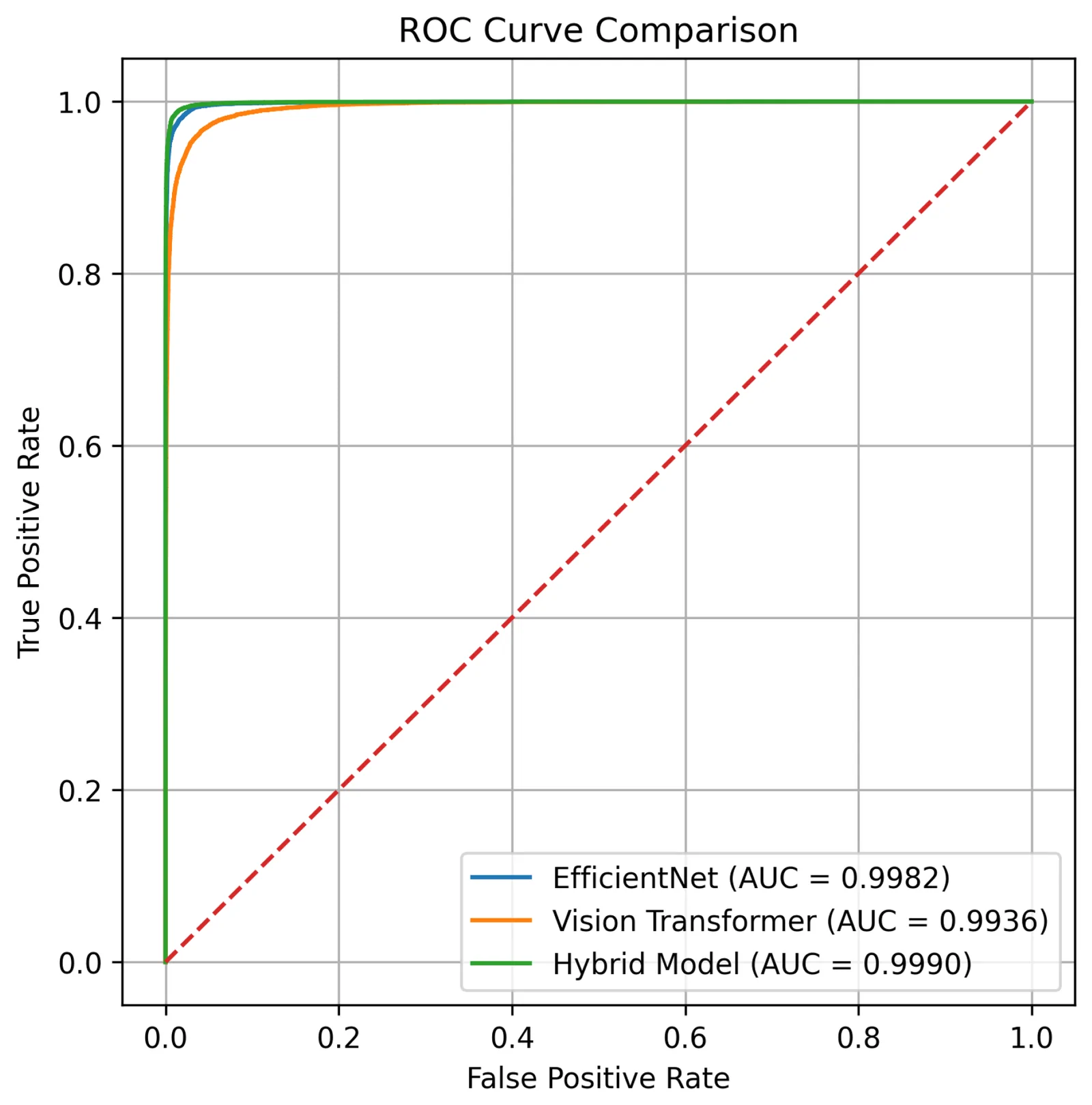
ROC curve comparison of the evaluated models.

Table 6 presents the performance evaluation of the proposed models. The Hybrid model achieved the best overall performance, obtaining an accuracy of 0.9870, a precision of 0.9852, a recall of 0.9889, and an F1-score of0.9871. Furthermore, it achieved the highest AUC value of 0.9990, indicating an excellent discrimination capability between real and manipulated images. EfficientNet also showed strong performance with an AUC of 0.9982 and an accuracy of 0.9754, while the Vision Transformer achieved an accuracy of 0.9619 and an AUC of 0.9936. Figure 7 compares the ROC curves of EfficientNet, Vision Transformer (ViT), and the proposed Hybrid model. These results indicate that the integration of CNN-based and Transformer-based features in the Hybrid architecture improves classification performance and provides the most reliable deepfake detection among the evaluated models.

### 5.1. Cross-Dataset Evaluation

To evaluate the generalization capability and robustness of the proposed framework across domain shifts, a cross-dataset evaluation was conducted. The model was trained exclusively on the deepfake and real images dataset and evaluated on an entirely unseen distribution, without any retraining or fine-tuning, the CelebDF-v2 Image Dataset [28].

**Figure 8:**
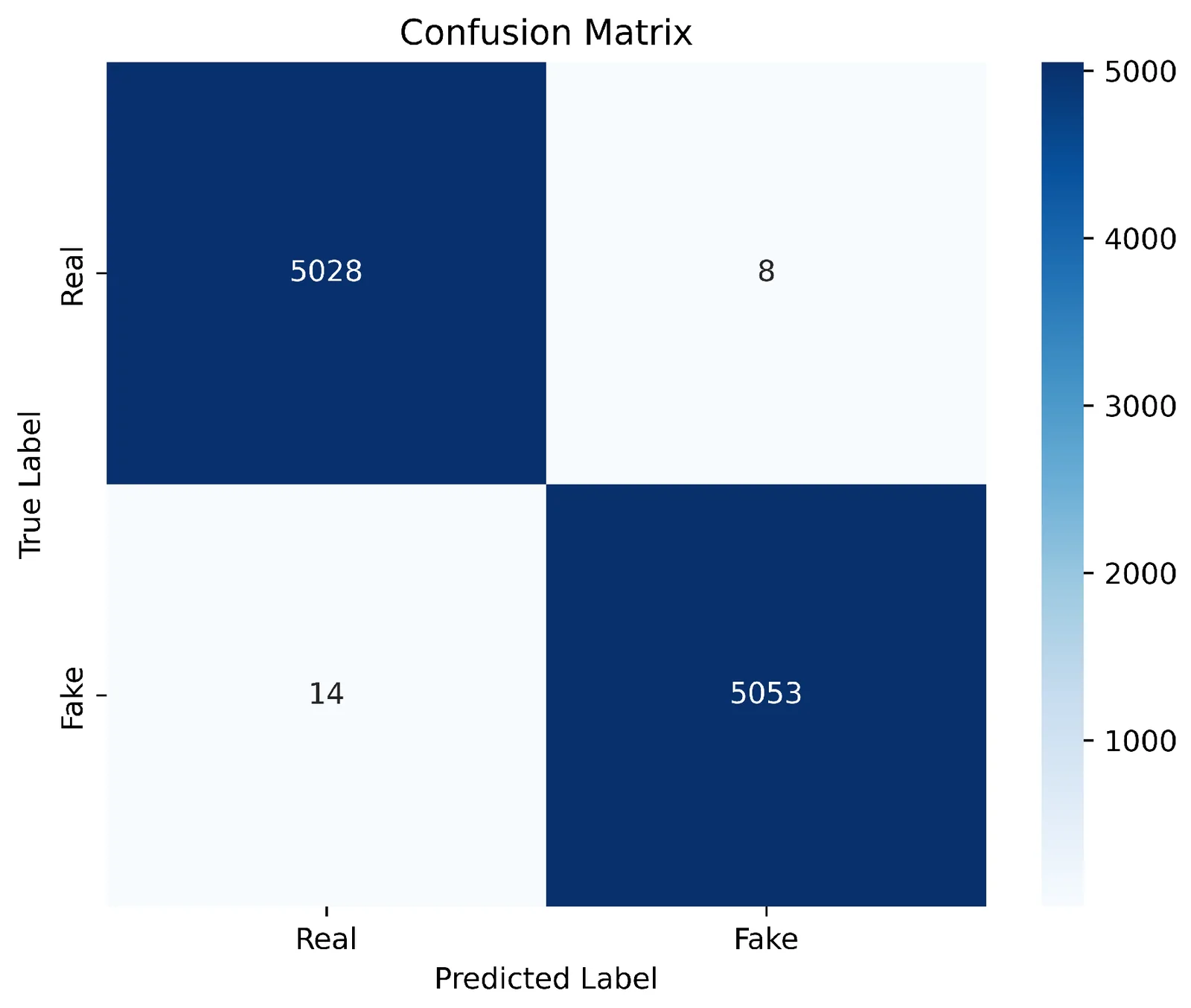
Confusion matrix obtained during cross-dataset evaluation on CelebDF-v2.

**Figure 9:**
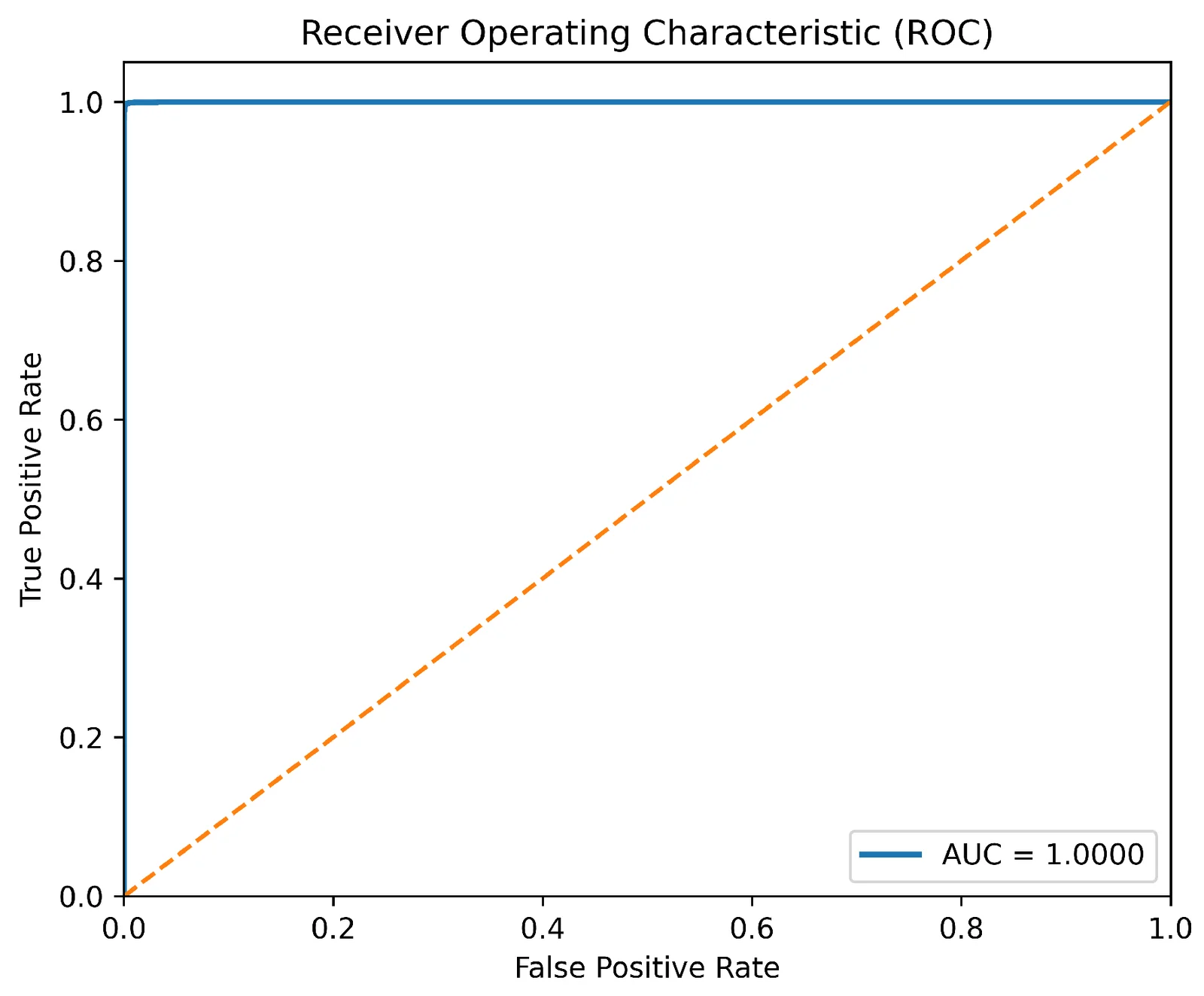
ROC curve of the proposed hybrid model on CelebDF-v2.

Table 7 shows the quantitative results obtained during the cross-dataset evaluation. Furthermore, the confusion matrix shown in Figure 8 demonstrates that only a very small number of samples were misclassified, while the ROC curve in Figure 9 confirms the excellent discriminative capability of the proposed framework. These results indicate that the hybrid EfficientNet-ViT architecture generalizes effectively to unseen datasets and remains highly reliable across different deepfake image distributions.

### 5.2. Robustness Against Realistic Image Degradations

To further investigate the robustness of the proposed hybrid model under practical deployment conditions, additional experiments were conducted by applying several common image degradations to the test images. Specifically, JPEG compression, Gaussian blur, Gaussian noise, and image resizing were introduced without performing any additional retraining or fine-tuning of the model.

To ensure reproducibility, the degradation parameters were fixed at severity level 1 for all experiments. JPEG compression was applied with a quality factor of 70. Gaussian blur was applied with a radius of 1, while Gaussian noise was added with zero mean and a standard deviation of 3 on the 0–255 image scale. For image resizing, the image dimensions were reduced to 85% of the original size using bilinear interpolation and then restored to the original dimensions using the same interpolation method. No additional retraining or fine-tuning was performed after applying these degradations.

Table 8 shows the obtained results. The proposed model consistently maintained excellent detection performance under all evaluated degradation types. The classification accuracy remained above 0.99 for all scenarios, while the AUC values were consistently close to 1.0000. These results demonstrate that the proposed EfficientNet–ViT hybrid architecture is highly robust to common image quality degradations frequently encountered in real-world image acquisition, transmission, and social media platforms.

These results demonstrate that the proposed framework maintains strong classification performance under the specific image degradations evaluated, indicating robustness to moderate image-quality perturbations.

### 5.3. Ablation Studies

To systematically analyze the contribution of the architectural components and regularization techniques in the proposed model, two ablation studies were conducted.

#### 5.3.1. Impact of Feature Fusion Strategy

To justify the selected feature fusion strategy, an ablation study was conducted by comparing the proposed feature concatenation approach with a simple averaging-based fusion mechanism. In both experiments, the same network architecture, training protocol, optimizer, learning rate, and dataset were used. The only difference was the feature fusion operation.

Table 9 demonstrates that the proposed feature concatenation strategy substantially outperforms simple averaging fusion. While averaging reduces the feature representations of both backbones into a single shared representation, feature concatenation preserves complementary information extracted by EfficientNet and Vision Transformer. Consequently, the proposed fusion strategy achieves significantly higher Accuracy, F1-score, and AUC, confirming that preserving both local and global feature representations is essential for reliable deepfake detection.

#### 5.3.2. Impact of Dropout Regularization

In addition, an ablation study was conducted to investigate the effect of dropout regularization on the proposed hybrid architecture. As shown in Table 10, the baseline model without dropout achieved an accuracy of 0.9870 and an AUC of 0.9990, whereas introducing a dropout layer (rate = 0.3) reduced the accuracy to 0.7072 and the AUC to 0.7938. These results indicate that dropout regularization is not beneficial for the proposed feature-fusion architecture. Since both EfficientNet-B0 and ViT-B/16 are initialized with pretrained weights and the training dataset is sufficiently large, the model already exhibits strong generalization capability. Additional dropout suppresses informative fused features, leading to a noticeable degradation in classification performance.

To place the proposed framework within the broader context of existing literature, Table 11 provides a qualitative literature-level comparison with several representative deepfake detection methods. It is important to clarify that this comparison serves as a high-level context overview rather than a direct benchmark on identical datasets or evaluation splits, as prior works frequently employ varying settings, dataset partitions, or video-level evaluations. Nevertheless, the proposed EfficientNet–ViT feature concatenation strategy demonstrates highly competitive performance, highlighting the effectiveness of integrating fine-grained spatial representations with global contextual dependencies. Direct metrics were not reported under comparable frame-level settings in the original publications. This table is intended solely as a literature-level reference, not a direct benchmark.

## 6. Conclusions and Future Work

In this study, we proposed a hybrid deep learning framework for image-level deepfake detection by combining EfficientNet-B0 and Vision Transformer (ViT-B/16). The proposed architecture exploits the complementary strengths of convolutional neural networks for extracting local visual features and transformer-based models for capturing global contextual relationships, resulting in a more discriminative feature representation for deepfake image classification.

Experimental results demonstrated that the proposed hybrid model outperformed the individual EfficientNet and ViT models, achieving an accuracy of 0.9870, an F1-score of 0.9871, and an Area Under the Curve (AUC) of 0.9990 on the deepfake and Real Images dataset. Furthermore, cross-dataset evaluation on the CelebDF-v2 image dataset confirmed the strong generalization capability of the proposed framework, achieving an accuracy of 0.9978 and an AUC of 1.0000 without modifying the network architecture. In addition, robustness experiments under common image degradations, including JPEG compression, Gaussian blur, Gaussian noise, and image resizing, demonstrated that the proposed model maintains consistently high detection performance under realistic image quality variations.

Overall, the experimental findings indicate that combining CNN-based local representations with transformer-based global representations provides a reliable and effective solution for deepfake image detection. The additional cross-dataset and robustness evaluations further support the practical applicability of the proposed framework in real-world scenarios.

As future work, we plan to evaluate the proposed framework on larger and more diverse deepfake datasets, investigate more advanced feature fusion strategies, and explore explainable artificial intelligence techniques to improve the interpretability of the model’s predictions and further enhance its robustness under challenging real-world conditions.

## Figures and Tables

**Table 1:** Summary of related work.

Reference	Method	Technique	Main Contribution
[12]	Head pose analysis	CNN	Detected abnormal head movements
[13]	Face warping detection	CNN	Identified face-swapping artifacts
[14]	Convolutional trace analysis	CNN	Detected fake image traces
[15]	Hybrid model	EfficientNet + ViT	Combined local and global features
[16]	M2TR	Multi-modal Transformer	Fused spatial and temporal features
[17]	Explainable detection	SHAP + DL	Improved model interpretability
[19]	Recurrent framework	CNN + RNN	Detected temporal inconsistencies
[20]	Two-branch network	RNN	Localized fake regions in videos
[21]	DFDC	Benchmark Dataset	Introduced large-scale dataset
[22]	FaceForensics++	Benchmark Framework	Standardized evaluation process
[23]	CNN-LSTM model	CNN + LSTM	Analyzed fake motion patterns
[24]	Capsule network	Capsule Networks	Captured hierarchical features
[25]	Sequential hybrid model	MLP + LSTM	Improved subtle manipulation detection

**Table 2:** Dataset partition and class distribution.

Subset	Total Images	Real	Fake	Ratio
Training	140,002	70,001	70,001	73.55%
Validation	39,428	19,787	19,641	20.72%
Testing	10,905	5413	5492	5.73%
Total	190,335	95,201	95,134	100%

**Table 3:** Implementation details of the proposed hybrid framework.

Parameter	Value
Framework	PyTorch 2.9.0+cu126
EfficientNet Backbone	EfficientNet-B0
Vision Transformer	ViT-B/16
Pretrained Weights	ImageNet
Input Image Size	224×224 pixels
Batch Size	32
Optimizer	Adam
Learning Rate	$1\times 10^{-4}$
Loss Function	Cross-Entropy Loss
EfficientNet Feature Dimension	1000
ViT Feature Dimension	1000
Fused Feature Dimension	2000
Classifier Head	Fully Connected Layer (2000 → 2)
Training Strategy	End-to-end Fine-tuning
Image Normalization	Mean = (0.5, 0.5, 0.5), Std = (0.5, 0.5, 0.5)

**Table 4:** Hardware and software specifications.

Component/Parameter	Specification
Platform	Kaggle Notebooks
Simulation Language	Python 3.12.12
Operating System	Windows 10
CPU	Intel Core i7
System RAM	16 GB
GPU Model	2 × NVIDIA Tesla T4
GPU VRAM	30 GB total (15 GB per GPU)
Disk Space	220 GB

**Table 5:** Hyperparameters used in model training.

Hyperparameter	Value
Learning Rate	0.0001
Optimizer	Adam
Loss Function	CrossEntropyLoss
Batch Size	32
Epochs	8
Learning-rate Schedule	None
Model Selection Criterion	Highest Validation Accuracy
Input Image Size	224 × 224
Model Architecture	EfficientNet + ViT (Hybrid)

**Table 6:** Performance evaluation of proposed models.

Model	Precision	Recall	F1-Score	Accuracy	AUC
EfficientNet (CNN)	0.9579	0.9948	0.9760	0.9754	0.9982
Vision Transformer (ViT)	0.9570	0.9676	0.9623	0.9619	0.9936
Hybrid (EfficientNet + ViT)	0.9852	0.9889	0.9871	0.9870	0.9990

**Table 7:** Cross-dataset evaluation results on the CelebDF-v2 image dataset.

Metric	Value
Accuracy	0.9978
Precision	0.9984
Recall	0.9972
F1-score	0.9978
AUC	1.0000

**Table 8:** Robustness evaluation under common image degradations.

Degradation	Accuracy	AUC
Clean Images	0.9978	1.0000
JPEG Compression	0.9931	0.9999
Gaussian Blur	0.9977	1.0000
Gaussian Noise	0.9976	0.9999
Image Resize	0.9950	0.9999

**Table 9:** Ablation study of different feature fusion strategies.

Fusion	Acc.	Prec.	Recall	F1-Score	AUC
Averaging	0.7177	0.7572	0.6348	0.6906	0.8002
Concatenation (Proposed)	0.9870	0.9852	0.9889	0.9871	0.9990

**Table 10:** Ablation study on the effect of dropout regularization.

Configuration	Accuracy	AUC
Without Dropout (Proposed)	0.9870	0.9990
With Dropout (Rate = 0.3)	0.7072	0.7938

**Table 11:** Literature-level comparison with representative deepfake detection methods.

Reference	Model	Year	Evaluation Setting/Dataset	Performance
[13]	Face Warping Artifacts	2018	DeepFake/FaceForensics	AUC = 0.843
[14]	Convolutional Trace Analysis	2020	Specific Synthesis Artifacts	N/A
[15]	EfficientNet + ViT	2022	DFDC/FaceForensics++	AUC = 0.951
[16]	M2TR (Multi-scale Transformer)	2022	FaceForensics++	Acc = 0.95
[23]	CNN-LSTM (Optical Flow)	2022	Video-level Sequences	Acc = 0.9121
[24]	Capsule Network	2019	Reenactment Scenarios	N/A
[25]	MLP-LSTM Hybrid Model	2023	Custom Frame Sequences	Acc = 0.7470
Proposed Work	EfficientNet + ViT Feature Fusion	2026	Deepfake-and-Real-Images (Frame-level)	Acc = 0.9870, AUC = 0.9990

## Data Availability

The raw data supporting the conclusions of this article will be made available by the authors on request.
